# Associations Between Transcranial Doppler Vasospasm and Clinical Outcomes After Aneurysmal Subarachnoid Hemorrhage: A Retrospective Observational Study

**DOI:** 10.7759/cureus.31789

**Published:** 2022-11-22

**Authors:** Abhijit V Lele, Rafael Wabl, Sarah Wahlster, Jade Keen, Andrew M Walters, Christine T Fong, Vasu B Dhulipala, Umeshkumar Athiraman, Anne Moore, Monica S Vavilala, Louis J Kim, Michael R Levitt

**Affiliations:** 1 Department of Anesthesiology and Pain Medicine, Harborview Medical Center, Seattle, USA; 2 Department of Neurology, Harborview Medical Center, Seattle, USA; 3 Department of Neurological Surgery, Harborview Medical Center, Seattle, USA; 4 Department of Anesthesiology and Pain Medicine, Washington University St.Louis, St.Louis, USA; 5 Department of Neurological Surgery, University of Washington, Seattle, USA

**Keywords:** outcomes, delayed cerebral ischemia, transcranial doppler, aneurysmal subarachnoid hemorrhage, ruptured intracerebral aneurysm, angiographic vasospasm, cerebral vasospasm

## Abstract

Objective: The objective is to examine the relationship between transcranial Doppler cerebral vasospasm (TCD-vasospasm), and clinical outcomes in aneurysmal subarachnoid hemorrhage (aSAH).

Methods: In a retrospective cohort study, using univariate and multivariate analysis, we examined the association between TCD-vasospasm (defined as Lindegaard ratio >3) and patient's ability to ambulate without assistance, the need for tracheostomy and gastrostomy tube placement, and the likelihood of being discharged home from the hospital.

Results: We studied 346 patients with aSAH; median age 55 years (Interquartile range IQR 46,64), median Hunt and Hess 3 [IQR 1-5]. Overall, 68.6% (n=238) had TCD-vasospasm, and 28% (n=97) had delayed cerebral ischemia. At hospital discharge, 54.3% (n=188) were able to walk without assistance, 5.8% (n=20) had received a tracheostomy, and 12% (n=42) had received a gastrostomy tube. Fifty-three percent (n=183) were discharged directly from the hospital to their home. TCD-vasospasm was not associated with ambulation without assistance at discharge (adjusted odds ratio, aOR 0.54, 95% 0.19,1.45), tracheostomy placement (aOR 2.04, 95% 0.23,18.43), gastrostomy tube placement (aOR 0.95, 95% CI 0.28,3.26), discharge to home (aOR 0.36, 95% CI 0.11,1.23).

Conclusion: This single-center retrospective study finds that TCD-vasospasm is not associated with clinical outcomes such as ambulation without assistance, discharge to home from the hospital, tracheostomy, and gastrostomy feeding tube placement. Routine screening for cerebral vasospasm and its impact on vasospasm diagnostic and therapeutic interventions and their associations with improved clinical outcomes warrant an evaluation in large, prospective, case-controlled, multi-center studies.

## Introduction

Delayed cerebral ischemia (DCI) is one of the strongest predictors of poor outcomes after aneurysmal subarachnoid hemorrhage (aSAH) [1]. DCI is believed to be multifactorial, with ischemia from large vessel vasospasm being the main potentially reversible clinical etiology. Therefore, the mainstay of the intensive care unit (ICU) management of aSAH patients focuses on detecting, preventing, and treating cerebral vasospasm.

Besides serial neurological assessments, the best screening modality for vasospasm has not been established. The recently proposed performance measure by the Neurocritical Care Society recommends that either CT angiography (CTA), digital subtraction angiography (DSA), or transcranial Doppler ultrasonography (TCD) may be performed between days 3 and 14 to screen for cerebral vasospasm in patients with aSAH [2]. Similarly, quality metrics from the American Heart Association do not specify the ideal frequency with which TCD, CTA, and DSA should be performed to diagnose cerebral vasospasm [3]. Correspondingly, the use of TCDs in aSAH varies amongst different aSAH treatment centers [4-6]. The heterogeneity largely persists because the relationship between symptomatic clinical vasospasm, TCD vasospasm (TCD-vasospasm), angiographic vasospasm on DSA, DCI, and functional outcomes is also unclear [7-12].

Given these uncertainties [11,13,14], we conducted a retrospective study of a large aSAH cohort at a major quaternary academic medical center with a high volume of aSAH to examine the association between TCD-vasospasm and clinical outcomes at hospital discharge, including ambulation without assistance, tracheostomy and gastrostomy placement, and the likelihood of discharge to home from the hospital. We tested the hypothesis that TCD-vasospasm is not associated with clinical outcomes at hospital discharge.

## Materials and methods

Institutional review board approval

The Institutional Review Board of the University of Washington approved this study (STUDY00004932) on March 3, 2020 and a waiver of consent was granted.

Study setting and patient selection

This retrospective cohort study was conducted on patients with aSAH admitted to Harborview Medical Center, a Comprehensive Stroke Center and high-volume aSAH center, between January 1, 2014 and December 31, 2019. Patients were included if they were 18 years or older, had a ruptured intracerebral aneurysm as the confirmed cause of their spontaneous SAH, underwent repair of a ruptured intracerebral aneurysm within the first 24 hours of admission, and received at least seven days of vasospasm screening using TCD. Patients with fewer than seven days of TCD examination were excluded since, at our institution, the standard duration of performing a TCD examination is typically 14 days.

Patient management

Patients with aSAH at our institution were managed according to the American Heart Association [15] and the Neurocritical Care Society [16] aSAH guidelines. All patients received nimodipine for 21 days and levetiracetam for seven days. Critical care management was focused on the maintenance of euvolemia, normonatremia (serum sodium > = 135-145 mEQ/L), normothermia (temperature 36.5-38.3 ºC), and euglycemia (blood glucose 100-180 mg/dL).

Screening and Management of Cerebral Vasospasm

TCD studies were performed by a dedicated team of technicians from the cerebrovascular laboratory. A complete TCD examination of the anterior and posterior circulation was performed once daily. The neurocritical care and neurosurgical team members discuss the results of TCD assessments during daily rounds. TCD data were incorporated into everyday clinical decision-making, such as induced hypertension, the performance of CTA or DSA, serial neurological assessments, and whether to pursue endovascular intervention in terms of intra-arterial vasodilator with or without balloon angioplasty. These practices are not protocolized but rather decided upon on a case-by-case basis. For example, CTA or DSA may be performed based on a change in clinical examination and or change in TCD data, such as worsening Lindegaard ratios (LR) or mean flow velocities, with prioritization given to a worsening of clinical examination as opposed to an isolated increase in TCD data without a clinical correlate.

Data collection

Data were collected via automated extraction and manual review from the electronic medical record. Baseline data included age, sex, Hunt and Hess (HH) grade, Fisher grade, location of the ruptured intracerebral aneurysm (anterior vs. posterior circulation), method of aneurysm repair (microsurgical vs. endovascular), placement of an external ventricular drain (EVD).

TCD examination data included 1) The number of TCD examinations, 2) maximal middle cerebral artery mean flow velocities (MCA-MFV), and 3) LR values (LR ratio = maximal mean flow velocity in the middle cerebral artery mean flow velocity/maximal mean flow velocity of the extracranial portion of the internal carotid artery). TCD-vasospasm was defined as LR > 3 [14]. We further categorized patients with LR>3 into mild-moderate TCD-vasospasm (LR >3<6) and severe TCD-vasospasm (LR >6). We calculated the maximum LR ratio (LRmax) and the difference between the highest and lowest LR (LRdelta) between post-bleed days 3-14. Maximal MCA-MFV were categorized as mild-moderate TCD-vasospasm (121-199 cm/sec) and severe TCD-vasospasm (>200 cm/sec). CTA examination data included whether a CTA was performed after the initial diagnostic CTA and the number of subsequent CTAs performed per patient. DSA data included whether a DSA was performed after the initial diagnostic/aneurysm repair DSA and whether endovascular vasospasm-treatment interventions such as balloon angioplasty or intra-arterial vasodilators were used. We also collected data on the number of DSA/per patient. DCI was defined based on the consensus recommendations by a group of experts in this field: “The presence of focal neurological impairment (such as hemiparesis, aphasia, apraxia, hemianopia, or neglect), or a decrease of at least 2 points on the Glasgow Coma Scale score, GCS (either on the total score or on one of its individual components [eye, motor on either side, verbal]), for at least one hour, and ruling out other confounding variables such as fever, electrolyte abnormalities, hydrocephalus, and seizures” [17].

Outcomes

The outcomes of interest were clinical outcomes such as the patient's ability to walk without assistance at discharge, placement of a tracheostomy and a gastrostomy tube, and discharge to home from the hospital. The ability of a patient to walk without assistance was abstracted from a manual review of notes from the physical therapist, occupational therapist, and bedside nurse. 

Statistical analysis

A descriptive analysis of the cohort characteristics is presented as counts and percentages. Continuous data normality was tested using the Shapiro-Wilk test. Age, LRmax, LRdelta, Hunt and Hess, and Fisher grade were expressed as median (IQR). Using the Wilcoxon test, we calculated the differences between the LRmax and MCA MFV among the four clinical outcomes of interest. We reported the 10-90th percentile of MCA-MFV and LRmax among the four clinical outcomes of interest. We also report the frequency of obtaining CTA, DSA, endovascular intervention, and induced hypertension in response to TCD-vasospasm presence and its severity. To test the association between TCD-vasospasm and patient outcomes, we performed univariate analyses testing for age, HH grade, Fisher score, location of the aneurysm, type of aneurysm repair (craniotomy vs. endovascular), placement of EVD, mechanical ventilation, TCD-vasospasm, LRmax, LRdelta, LR >3<6, LR >6, MCA-MFV 120-199 cm/sec, MCA MFV >200 cm/sec, DCI with each outcome of interest. We excluded mechanical ventilation status from the univariate analysis for tracheostomy placement. A multivariate regression model then tested the association between significant factors found in the univariate model and each patient outcome (discharge to home, ambulation without assistance, tracheostomy, and gastrostomy feeding tube). The multivariate model for discharge to home was adjusted for age, HH score, Fisher grade, EVD, mechanical ventilation, DCI, and cerebral infarction. The model for ambulation without assistance was adjusted for age, HH score, EVD, mechanical ventilation, DCI, and cerebral infarction. The model for tracheostomy placement was adjusted for HH score, microsurgical repair, and cerebral infarction. The model for PEG placement adjusted for age, HH score, Fisher, mechanical ventilation, DCI, and cerebral infarction. We performed the Hosmer-Lemeshow goodness-of-fit test for all the multivariate models, and the results show that the data fit the model. Results were presented as the adjusted odds ratio or aOR and the corresponding 95% confidence interval. A p-value of < 0.05 indicated statistical significance. STATA 15.0 [18]/R studio, version 4.1.1 [19], was used for statistical analysis.

## Results

Cohort description

From an initial cohort of patients with aSAH (n=454) who underwent aneurysm repair, 85 patients were excluded because their aneurysm was repaired more than 24 hours after admission. Another 23 were excluded as they received TCD assessments for fewer than seven days. Three hundred forty-six patients were included in the final analysis. Cohort characteristics by TCD-vasospasm status are provided in Table 1. Most patients were female (71.1%, n=246) with a ruptured aneurysm in the anterior circulation (81%, n=281). Fifty-one percent (n=176) underwent microsurgical repair, and the majority (84%, n=291) received an EVD. Of 128 patients that received an MRI, 81 (63.3%) had cerebral infarction on MRI; cerebral infarction was observed in 63 (62%) patients with TCD-vasospasm and in 18 (69%) patients without TCD-vasospasm. At hospital discharge, 54.3% (n=188) were able to walk without assistance, 5.8% (n=20) had received a tracheostomy, and 12% (n=42) had received a gastrostomy tube. Fifty-three percent (n=183) were discharged directly from the hospital to their home.

**Table 1:** Study cohort characteristics by transcranial Doppler vasospasm status IQR: interquartile range; LR: Lindegaard ratio

	Overall, N = 346	No TCD Vasospasm, N = 108	TCD Vasospasm, N = 238
Age in Years Median (IQR)	55 (46, 64)	62 (51, 68)	52 (43, 59)
Sex			
Female	246(71.1%)	84(34.2%)	162 (65.8%)
Male	100(29%)	24(24%)	76(76%)
Hunt and Hess Grade Median (IQR)	3.00 [1.00, 5.00]	﻿3.00 [1.00, 5.00]	3.00 [1.00, 5.00]
Fisher Grade Median (IQR)	3.00 [1.00, 4.00]	﻿3.00 [1.00, 4.00]	3.00 [1.00, 4.00]
Ruptured Aneurysm in the Anterior circulation	281 (81%)	89 (82%)	192 (81%)
External Ventricular Drain	291 (84%)	85 (79%)	206 (87%)
Microsurgical Cerebral Aneurysm Repair	176 (51%)	38 (35%)	138 (58%)
Endovascular Cerebral Aneurysm Repair	170 (49%)	70 (65%)	100 (42%)
Mechanically Ventilated	201 (58%)	46 (43%)	155 (65%)
Ambulate Without Assistance	188 (54%)	62 (57%)	126 (53%)
Discharged Home	183 (53%)	61 (56%)	122 (51%)
Tracheostomy Placement	20 (5.8%)	3 (2.8%)	17 (7.1%)
Gastrostomy Feeding Tube Placement	42 (12%)	12 (11%)	30 (13%)
Maximum Lindegaard Ratio (LRmax)	3.82 (2.80, 5.13)	2.50 (2.26, 2.78)	4.54 (3.76, 5.70)
Change in Lindegaard Ratio from Baseline (LRdelta) Median (IQR)	1.60 (0.76, 2.99)	0.67 (0.27,1.00)	2.44 (1.50,3.45)
Total Number of Transcranial Doppler Examinations Median (IQR)	14 (13, 15)	13 (12, 14)	14 (13, 16)
Total Number of Computed Tomography Angiography Examinations			
0	195 (56%)	85 (79%)	110 (46%)
1	85 (25%)	15 (14%)	70 (29%)
2	44 (13%)	7 (6.5%)	37 (16%)
3	14 (4.0%)	1 (0.9%)	13 (5.5%)
4	6 (1.7%)	0 (0%)	6 (2.5%)
5	2 (0.6%)	0 (0%)	2 (0.8%)
Total Number of Digital Subtraction Angiography Examinations			
0	283 (82%)	99 (92%)	184 (77%)
1	58 (17%)	8 (7.4%)	50 (21%)
2	5 (1.4%)	1 (0.9%)	4 (1.7%)
Intensive Care Unit Length of Stay in Days Median (IQR)	15 (12, 17)	13 (10, 15)	16 (13, 18)
Hospital Length of Stay in Days Median (IQR)	20 (15, 29)	18 (13, 26)	22 (16, 30)

TCD screening for vasospasm****

*Frequency of TCD Screening* 

Patients received a median of 14 [IQR 2] TCD examinations each. The median number of TCD examinations was higher in patients with TCD-vasospasm; 14 (13, 16) days vs. 13 (12, 14) days, p-value <0.001. The total number of TCD examinations was higher in patients with LRmax >6 (median 16, IQR 3) than in patients with LRmax >3<6 (median 14, IQR 2).

Prevalence of Cerebral Vasospasm and Further Testing and Interventions

TCD-vasospasm was observed in 238 patients (63.3%). 56.6% (n=196) had 6>LR>3, while 13% (n=45) had LR >6. The median LRmax was 3.82 [2.8, 5.13], and the median LRdelta was 1.6 [0.76, 2.99]. Overall, 43.9% (n=152) had MCA-MFV velocities of 120-199 cm/sec, while 18.7% (n=65) had MCA MFV velocities > 200 cm/sec.

Among the entire cohort of 346 patients, 151 (43.6%) patients underwent CTA, and 97 (28%) underwent DSA. Of note, 190 (54.9%) had a TCD + CTA + DSA, 97 (28%) had TCD + CTA, 38 (10.9%) had TCDs only, and 21 (6%) had a TCD + DSA. Overall, 128/238 patients who had TCD-vasospasm went on to receive CTA, 81/238 (34%) went on to receive a DSA, 54/238 (22.7%) received an end-vascular intervention, while 17/238 (7%) had induced hypertension.

Amongst all patients, 64 (18.4%) patients underwent endovascular VSPM intervention; 30 (8.7%) balloon angioplasty, 25 (7.2%) combined balloon angioplasty and intra-arterial vasodilator, and nine (2.6%) received an intra-arterial vasodilator. In contrast, the remaining 33 patients who underwent a DSA did not receive any intervention. The relative risk of getting a DSA when a patient had received a CTA was 3.92, 95%CI 2.61,5.91.

**Figure 1:**
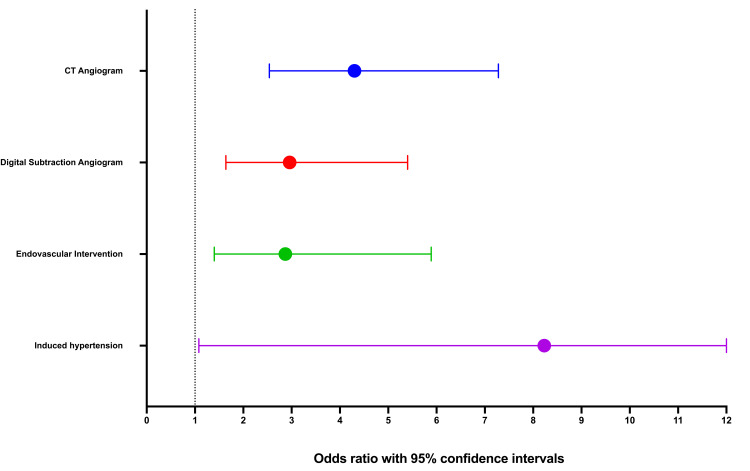
Diagnostic and therapeutic interventions in patients with transcranial Doppler vasospasm CT: computerized tomography of the head and neck

As shown in Table 1, 58 patients (24.4%) with TCD-vasospasm underwent more than one CTA, and five (1.4%) underwent more than one DSA examination. TCD-vasospasm was associated with increased rates of CTA (odds ratio, OR 4.3, 95% CI 2.54,7.28), DSA (OR 2.96, 95% CI 1.64,5.40), and an increased likelihood of endovascular vasospasm intervention (OR 2.87, 95% CI 1.40,5.89) and induced hypertension (OR 8.23, 95% CI 1.08,62.7) as highlighted in Figure 1.

Similarly, DCI was also associated with the performance of DSA (OR 3.5, 95% CI 2.11-5.79), endovascular intervention: OR 3.93, 95% CI 2.23-6.93), CTA (OR 2.64, 95% 1.63-4.29), and induced hypertension (OR 2.14, 95% CI 0.82-5.6).

Association between TCD vasospasm and clinical outcomes

**Figure 2:**
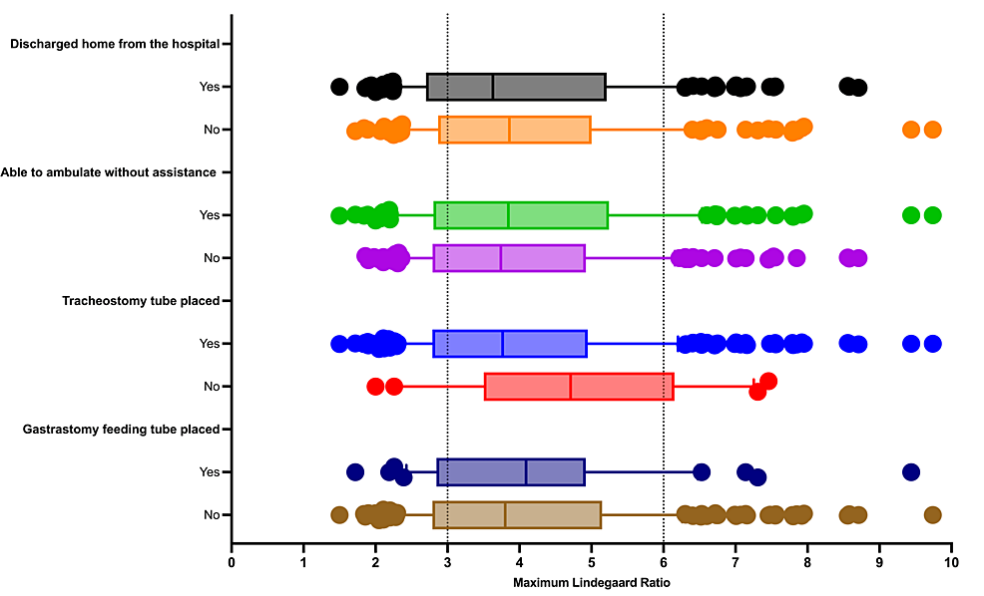
Differences in maximum Lindegaard ratios amongst the clinical outcomes of interest reference lines at 3 and 6 divide the Lindegaard ratios into mild-moderate (LR >3<6) and severe (LR >6) categories Note: Differences between clinical outcome groups are not statistically significant

**Figure 3:**
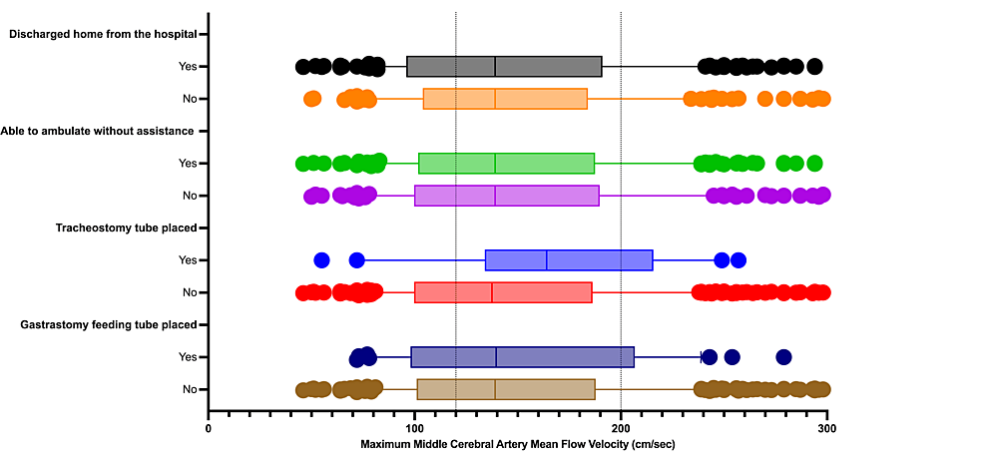
Differences in the maximum middle cerebral artery mean flow velocities amongst the clinical outcomes of interest Reference lines at 120 cm/sec and 200 cm/sec divide the velocities into mild-moderate (<120<200 cm/sec) and severe (>200 cm/sec) categories. Note: Differences between clinical outcome groups are not statistically significant

The differences between the LRmax and the maximal MCA-MFV amongst the clinical outcome groups are demonstrated in Figures 2, 3. We did not find any statistically significant differences between the LRmax and MCA MFV among the four clinical outcomes of interest.

Univariate Analysis

The results of the univariate analysis are presented in Table 2. Of note, TCD-vasospasm was not associated with discharge to home (OR 0.81, 95% CI 0.51,1.28, AUC 0.52), ambulation without assistance (OR 1.2, 95% CI 0.76,1.9, AUC 0.52), tracheostomy (OR 2.69, 95% CI 0.77,9.39, AUC 0.58), or gastrostomy placement (OR 1.15, 95% CI 0.57,2.35, AUC 0.52). LRmax >3<6 and LRmax >6 were not associated with discharge to home, ambulation without assistance, tracheostomy, or gastrostomy feeding tube (Table 2).

**Table 2:** Univariate analysis of the factors associated with outcomes of interest AUC = area under the curve MCA-MFV: Middle cerebral artery mean flow velocities Definitions: Delayed Cerebral Ischemia: “The occurrence of focal neurological impairment (such as hemiparesis, aphasia, apraxia, hemianopia, or neglect), or a decrease of at least 2 points on the Glasgow Coma Scale (either on the total score or on one of its components [eye, motor on either side, verbal]). This should last for at least 1 hour, is not apparent immediately after aneurysm occlusion, and cannot be attributed to other causes utilizing clinical assessment, CT or MRI scanning of the brain, and appropriate laboratory studies.” [16] Transcranial Doppler Vasospasm: Lindegaard ratio > 3

	Discharge home	Ambulate without assistance	Tracheostomy	Gastrostomy feeding tube
Age in years	OR 0.96 (0.94,0.97) AUC 0.65	OR 0.96 (0.95,0.98) AUC 0.63	OR 1.01 (0.96,1.06) AUC 0.58	OR 1.08 (1.05,1.11) AUC 0.75
Hunt and Hess Score	OR 0.38 (O.28,0.49) AUC 0.74	OR 0.64 (0.52,0.8) AUC 0.62	OR 1.85 (1.16,2.97) AUC 0.66	OR 1.95 (1.38,2.75) AUC 0.67
Fisher Score	OR 0.56 (0.38,0.82) AUC 0.57	0.77 (0.52,1.16) AUC 0.51	OR 1.21(0.55,2.66) AUC 0.51	OR 1.42 (0.80,2.52) AUC 0.54
External Ventricular Drain	OR 0.20 (0.1,0.41) AUC 0.59	OR 0.31 (0.16,0.60) AUC 0.57	OR 1.74(0.39,7.75) AUC 0.53	OR 2.68 (0.79,9.01) AUC 0.55
Mechanical Ventilation	OR 0.17 (0.11,0.27) AUC 0.69	OR 0.51 (0.32,0.78) AUC 0.58	NA	OR 6.31 (2.41,16.5) AUC 0.67
Anterior circulation aneurysm	OR 1.01 (0.60,1.76) AUC 0.50	OR 0.81 (0.47,1.40) AUC 0.51	OR 0.40 (0.15,1.05) AUC 0.58	OR 0.82 (0.38,1.83) AUC 0.51
Microsurgical Repair of the Ruptured Aneurysm	OR 0.65 (0.42,0.1) AUC 0.55	OR 0.96 (0.63,1.47) AUC 0.50	OR 2.92 (1,03,8.24) AUC 0.62	OR 1.13 (0.59,2.17) AUC 0.52
Endovascular Repair of the Ruptured Aneurysm	OR 1.45 (0.95,2.22) AUC 0.52	OR 1.07 (0.70,1.64) AUC 0.51	OR 0.41 (0.16,1.10) AUC 0.52	OR 0.81 (0.42,1.56) AUC 0.52
Delayed Cerebral Ischemia	OR 0.23 (0.14,0.38) AUC 0.64	OR 0.45 (0.28,0.73) AUC 0.58	OR 2.21 (0.89,5.52) AUC 0.59	OR 6.02 (3.03,11.94) AUC 0.71
Transcranial Doppler Vasospasm	OR 0.81 (0.51,1.28) AUC 0.52	OR 1.2 (0.76,1.9) AUC 0.52	OR 2.69(0.77,9.39) AUC 0.58	OR 1.15 (0.57,2.35) AUC 0.52
Maximal Lindegaard Ratio	OR 0.94 (0.83,1.08) AUC 0.53	OR 0.69 (0.23, 2.1) AUC 0.51	OR 1.28 (0.99,1.65) AUC 0.63	OR 1.04 (0.86,1.28) AUC 0.53
Change in Lindegaard Ratio from Baseline	OR 0.98 (0.85,1.12) AUC 0.51	OR 0.93 (0.34,2.53) AUC 0.50	OR 1.23 (0.94,159) AUC 0.59	OR 1.02 (0.83,1.25) AUC 0.50
Lindegaard ratio > 3 < 6	OR 0.76(0.49,1.18) AUC 0.53	OR 0.97(0.64,1.50) AUC 0.51	OR 1.16(0.46,2.91) AUC 0.52	OR 1.28 (0.66,2.48) AUC 0.53
Lindegaard ratio > 6	OR 1.25(0.66,2.36 AUC 0.51	0.86(0.46,1.610 AUC 0.51	OR 2.38(0.83,6.91) AUC 0.56	OR 0.89(0.33.2.40) AUC 0.50
MCA MFV >120<200 cm/sec	OR 0.89 (0.58,1.37) AUC 0.51	OR 1.24(0.81,1.89) AUC 0.53	OR 1.33(0.54,3.28) AUC 0.53	OR 0.62(0.32,1.22) AUC 0.56
MCA MFV > 200 cm/sec	OR 0.97(0.56,1.67) AUC 0.50	OR 0.77(0.45,1.33) AUC 0.52	OR 1.94(0.71,5.27) AUC 0.56	OR 1.64(0.78,3.37) AUC 0.54
Cerebral infarction on MRI	OR 0.49(0.23,1.057) AUC 0.58	OR 0.76(0.36,1.57) AUC 0.53	OR 1.87(0.48,7.29) AUC 0.57	OR 3.21(1.12,9.14) AUC 0.62

Multivariate Analysis 

**Figure 4:**
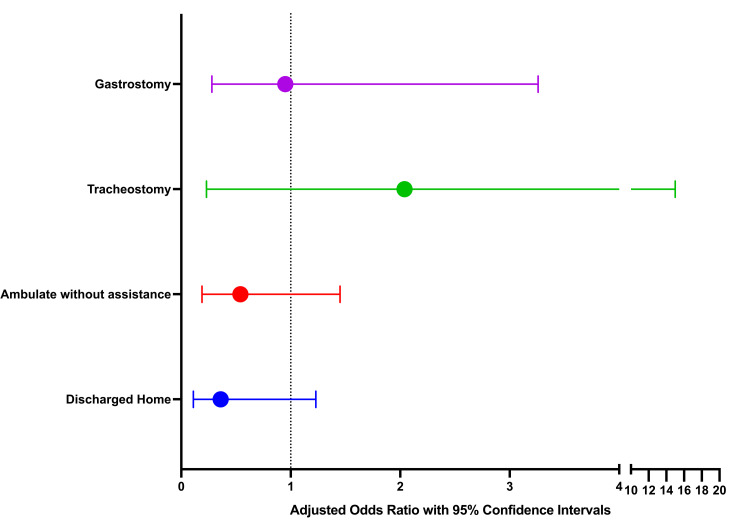
The association between transcranial Doppler vasospasm and clinical outcomes: result of multivariate analysis

The results of the multivariate analysis are presented in Figure 4. After adjusting for all significant variables with a p-value of < 0.05 in the univariate analysis, TCD-vasospasm was not associated with discharge to home (aOR 0.36, 95% CI 0.11,1.23), ambulation without assistance (aOR 0.54, 95% CI 0.19,1.45), tracheostomy (aOR 2.04, 95% CI 0.23,18.43), and gastrostomy feeding tube placement (aOR 0.95, 95% CI 0.28,3.26).

## Discussion

This single-center retrospective cohort study examined the association between TCD-vasospasm and clinical outcomes after aSAH. The main findings of this study are that TCD-vasospasm was not associated with discharge to home, ambulation without assistance, tracheostomy, or gastrostomy feeding tube placement.

The clinical outcome measures (ability to be discharged home from the hospital, ambulating without assistance, tracheostomy placement, and gastrostomy tube placement) used in this study have not previously been reported associated with TCD-vasospasm. The finding that TCD-vasospasm may not be related to clinical outcomes adds to the growing literature regarding the poor association between the two [7]. It is not surprising that the diagnosis of TCD-vasospasm resulted in further diagnostic testing and intervention since this is our clinical workflow and similar to previously published studies since the diagnosis of DCI may not always be clear, especially in comatose patients, which may prompt further investigations [20,21].

There may be many explanations for this: TCD imaging is performed at a single time point and may not accurately capture the physiology of the intracranial vasculature over the entire day. TCDs are also technically difficult studies to perform and thus highly operator dependent. In addition, the connection between angiographic vasospasm and DCI is tenuous from the outset; using TCDs for screening and including asymptomatic patients likely further degrades its association with meaningful outcomes. This lack of correlation with clinical outcomes requires us to carefully consider the reasons for routinely using TCD as a screening tool for cerebral vasospasm. While historically, trends in TCD data have been used to alert clinicians of impending symptomatic vasospasm [22], DCI may be a more clinically meaningful definition than the presence of arterial spasm by angiography or TCD [7]. In a study based out of the United Kingdom, Hollingworth et al. observed that centers that screened for vasospasm using TCD had worse in-hospital outcomes but similar rates of DCI than centers that did not screen with TCD [23]. Whether TCD data allow clinicians to intervene before clinical vasospasm and thus prevent long-term adverse neurological sequelae needs detailed examination in future prospective studies.

The fundamental question of whether TCD screening makes a difference in patient outcomes remains unanswered. The best way to address this is to compare patients who received TCD-based assessments to those patients who did not. Our study cannot answer this question directly due to the lack of a control group. However, the study that comes closest is the one published by Ehrlich et al. [14]. Using an exploratory model (50 patients with daily TCDs, compared to 39 patients without daily TCD), the investigators demonstrated no difference in clinical outcomes (mortality, Glasgow Outcome Scale, modified Rankin scale, and National Institute of Health Stroke Scale) between the two groups after aSAH. Patients with vasospasm are inherently expected to do worse. Thus, we cannot say that TCD monitoring did nothing to patients included in our study, but it may equally be that screening and intervening led to equivalent outcomes to those without vasospasm.

With the Neurocritical Care Society’s recent call for routine screening for vasospasm with TCD, CTA, or DSA as a potential performance measure, there is a new imperative to delineate the cost-benefit trade-off to patients and the hospital systems at large of cerebral vasospasm screening. Since practices for vasospasm screening vary widely [4], large, multicenter, prospective studies are warranted to generate high-quality evidence to establish a standard of care. Such studies may afford us opportunities to study the quality-of-care delivery patterns, such as techniques for screening cerebral vasospasm, adherence to the definition of DCI in daily clinical examination that may trigger further testing or intervention, routine performance of MRI to detect cerebral infarction and examine differences in clinical outcomes in patients subjected to further diagnostic testing and therapeutic interventions.

Limitations

The limitations of this study include its retrospective design, wherein the association between TCD-vasospasm and outcomes may depend on local practice. In addition, our population size, while an extensive aSAH database, is too small to have significant power to detect any small but potentially meaningful contribution of TCD-vasospasm. The estimated sample size is more than 1,000 patients. While most retrospective cohort studies analyzing TCDs are confounded by indication, this study, in addition to serial neurological clinical assessments, used TCDs as a screening methodology and avoided this bias. The study did not compare a non-monitored group; that would be the only way to show that TCD monitoring really helps or does not help. The outcome measures were assessed by individuals not blinded to TCD data.

## Conclusions

This single-center retrospective study finds that TCD-vasospasm is not associated with clinical outcomes such as ambulation without assistance, discharge to home from the hospital, tracheostomy, and gastrostomy feeding tube placement. Routine screening for cerebral vasospasm and its impact on further diagnostic and therapeutic interventions and their associations with improved clinical outcomes warrant an evaluation in large, prospective, case-controlled, multi-center studies.
